# Low-Dose-Area-Constrained Helical TomoTherapy-Based Whole Breast Radiotherapy and Dosimetric Comparison with Tangential Field-in-Field IMRT

**DOI:** 10.1155/2013/513708

**Published:** 2013-08-14

**Authors:** Jie Qiu, Zhikai Liu, Bo Yang, Xiaorong Hou, Fuquan Zhang

**Affiliations:** Peking Union Medical College Hospital, No. 1 Shuaifuyuan, Dongcheng District, Beijing 100730, China

## Abstract

*Background and Purpose.* To present a novel helical TomoTherapy-based method for whole breast radiotherapy that has better dosimetry and also has acceptable low-dose regions for lungs, heart, and contralateral breast compared with tangential field-in-field IMRT (FIF-IMRT). *Material and Methods.* Ten patients with left-side breast cancer were planned with low-dose-area-constrained helical TomoTherapy (LDC-HT) and FIF-IMRT. Dosimetry was compared for all techniques. *Results.* Coverage of the whole breast was adequate with both techniques. Homogeneity index (HI) and conformity index (CI) were better with LDC-HT. LDC-HT showed dosimetry advantages over FIF-IMRT for ipsilateral lung and heart in not only high-dose levels but also in low-dose levels such as *V*_10 Gy_ and *V*_5 Gy_. For contralateral lung, both techniques can provide good protection, although the mean dose of LDC-HT is higher than that of FIF-IMRT. *Conclusions.* With LDC-HT, we obtained adequate target coverage, better HI and CI of target volume, better sparing of organs at risk, and acceptably low-dose areas compared with FIF-IMRT. LDC-HT could be a feasible method in whole breast radiotherapy. Clinical benefits of LDC-HT need further investigation.

## 1. Introduction

Adjuvant whole breast radiotherapy following breast conserving surgery is the standard of treatment for early-stage breast cancer because it improves local control rates over breast-conserving surgery alone [1]. The standard technique for whole breast irradiation has been the delivery of two tangential fields, an approach which has not significantly changed for many decades.

Tangential 3D-CRT and field-in-field IMRT (FIF-IMRT) are commonly used tangential techniques. FIF-IMRT for whole breast irradiation is based on a standard tangential beam arrangement, employing two directly opposed fields. Subfields are added using forward or inverse planning to even out volumes of high and low doses throughout the whole breast volume; subfields are not usually used to spare the organs at risk, that is, the heart and lungs. Compared with 3D-CRT, FIF-IMRT improves dose homogeneity in whole breast radiotherapy, which correlates with less acute skin and soft tissue toxicities and better cosmesis of the treated breast in the long term [2]. But doses to heart and lungs are not significantly improved compared with 3D-CRT.

Multifield intensity-modulated radiotherapy (MF-IMRT) could achieve superior dose homogeneity and normal tissue sparing and has been applied to many tumors. It involves more complex beam arrangements and more beam angles than FIF-IMRT. High-dose regions of ipsilateral lung and heart for tumors of the left breast can be reduced compared with FIF-IMRT [3]. However, MF-IMRT is associated with an increased low-dose area, especially for organs not normally irradiated in conventional tangential radiotherapy, such as the contralateral lung and breast. Helical TomoTherapy (HT) delivers a kind of MF-IMRT. In HT treatment, a gantry continuously rotates around the patient while the patient is translated through the beam delivery plane. Hundreds of beamlets, created by passing a fan-beam through a high-speed binary collimator, can be delivered from any gantry angle. The use of all gantry angles might result in a large low-dose area in the body that would normally receive only scatter dose. The significance of this low-dose “bath” is unknown, although it is the main concern regarding late oncogenesis. The purpose of this study is to provide a new method of helical TomoTherapy-based whole breast radiotherapy which minimizes the low-dose area.

## 2. Patients and Methods

### 2.1. Patients and Planning Images

In this study, the sample comprised 10 left-side breast cancer patients treated with breast conserving surgery and whole breast radiotherapy at Peking Union Medical College Hospital, China, in 2011-2012. All patients underwent 5 mm slice thickness computer tomography (CT) scanning in the supine position on an inclined breast board with both arms abducted above the head. Images were acquired from the lower part of neck to 5 cm below the lowest part of the breast, with radiopaque wires marking the clinically detectable ipsilateral breast borders.

### 2.2. Treatment Volumes and Organs at Risk

The clinical target volume (CTV) was the whole ipsilateral breast delimited within the radiopaque wires. The planning target volume (PTV) was generated by adding an 8 mm margin round CTV to allow for respiratory motion and setup errors but confined to the interior of the body outer contours reduced by 5 mm. Organs at risk (OARs) contoured were both lungs, heart, and contralateral breast.

### 2.3. Dose Goals

The prescribed dose fractionation for the whole breast was 46 Gy in 2 Gy daily fractions over 4-5 weeks. We required that 95% of PTV receive 95% to 105% of the prescribed dose. The treatment volume receiving more than 107% of the prescribed dose should be less than 1%. The volume of ipsilateral lung receiving more than 40 Gy (*V*_40 Gy_), 30 Gy (*V*_30 Gy_), 20 Gy (*V*_20 Gy_), and 5 Gy (*V*_5 Gy_) should be as low as possible. The volume of heart receiving more than 30 Gy (*V*_30 Gy_) and that of the contralateral lung receiving more than 5 Gy (*V*_5 Gy_) were also minimized.

### 2.4. FIF-IMRT Planning

FIF-IMRT plans were also created with Eclipse version 8.0 treatment planning software. FIF-IMRT plans used the same energies and beam angles as 3D-CRT plans. Inverse planning was used to minimize the volume receiving higher than 107% of the prescribed dose.

### 2.5. HT Planning

HT plans were created with TomoTherapy treatment planning software. HT plans were delivered with 6MV X-rays. HT plan parameters consisted of a 2.5 cm field width (FW), 0.287 pitch, and a modulation factor (MF) of 3.0. 

A new support organ was defined for each patient as follows. A tangential line were drawn at the posterior border of traditional 3D-CRT beam, and the ipsilateral lung and heart below this line were defined as a new structure “block 1.” The structure “block 1” was designated as “completely blocked.” Contralateral lung and contralateral breast were designated as “directionally blocked.”

### 2.6. Data and Statistical Analysis

Dosimetric data was extracted from each planning system. Student's *t*-tests were used to compare data between LDC-HT and FIF-IMRT. Differences were considered significant for *P* < 0.05. 

## 3. Results

### 3.1. Patient Characteristics

**Figure 1:**
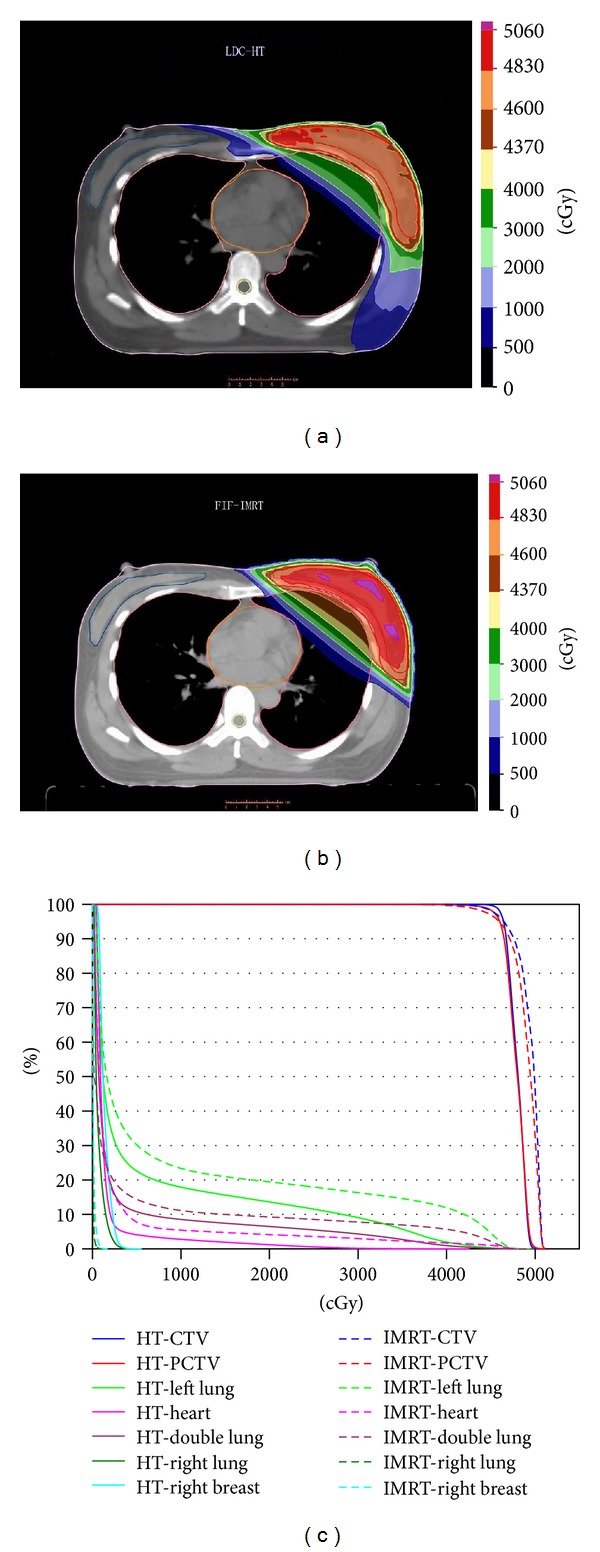
Isodose and cumulative DVHs for each technique.

Ten patients with left-sided breast cancer were selected for this study. PTV size varied from 424 cm^3^ to 885 cm^3^, with a mean of 630 cm^3^ and a median of 650 cm^3^. PTV and organs at risk (OARs) cumulative dose volume histograms (DVHs) and isodose distributions for a typical left-sided breast plan are illustrated in Figure 1.

### 3.2. Target Volume

The average dosimetric characteristics of the target for both techniques were presented in Table 1. Target coverage was adequate in all patients for both LDC-HT and FIF-IMRT. Mean volumes receiving at least 95% of prescribed dose for both techniques were similar (98.73% versus 98.53%; *P* = 0.392). Mean *V*_110%_ seemed better for LDC-HT than FIF-IMRT but not significantly (0.7% versus 15.68%; *P* = 0.056). Mean target doses were significantly lower for LDC-HT (48.28 Gy versus 49.18 Gy; *P* = 0.002), but maximum doses were not different (51.47 Gy versus 51.36 Gy; *P* = 0.667). Homogeneity index (1.08 versus 1.10; *P* = 0.001) and conformity index (0.83 versus 0.76; *P* = 0.023) were better for LDC-HT than FIF-IMRT.

### 3.3. Organs at Risk (OARs)

The average dosimetric characteristics of the organs at risk for both techniques were presented in Table 2. LDC-HT provided significant decreases in *V*_5 Gy_ (28% relative decrease), *V*_10 Gy_ (30% decrease), *V*_20 Gy_ (35% decrease), *V*_30 Gy_ (46% decrease), *V*_40 Gy_ (61% decrease), and mean dose (32% decrease) for ipsilateral lung. Similar results were obtained for both lungs combined. Both techniques provided very low doses to the heart, but LDC-HT was better for the heart *V*_5 Gy_ (57% relative decrease), *V*_10 Gy_ (59% decrease), *V*_20 Gy_ (71% decrease), *V*_30 Gy_ (82% decrease), *V*_40 Gy_ (91% decrease), and mean dose (45% decrease). Both techniques yielded very low doses to the contralateral lung and contralateral breast with maximum doses less than 5 Gy. However, FIF-IMRT resulted in lower maximum and mean doses for both contralateral lung and contralateral breast.

### 3.4. Treatment Time

Average treatment time for LDC-HT was 718.5 s (range: 635.7–835.4 s). The treatment time did not account for the patient setup and MVCT scan time. We estimated the total treatment time per patient to be approximately 20–25 min for LDC-HT. The average beam on time for FIF-IMRT was 357.6 s (range: 309.0–391.0 s). We thus estimated the total treatment time per patient to be 12–15 min for FIF-IMRT.

## 4. Discussion

Whole breast radiotherapy with boost following breast-conserving surgery is the standard of care for early-stage breast cancer patients. The 2-year local control rate could be higher than 90% [4]. And radiotherapy reduced the 10-year risk of any (i.e., locoregional or distant) first recurrence from 35.0% to 19.3% [5]. The main concern of whole breast radiotherapy now is the reduction of patient toxicity. The tangential technique for whole breast radiotherapy has not significantly changed over many decades. FIF-IMRT in this study is a type of tangential technique. FIF-IMRT could reduce maximum target doses relative to 3D-CRT. 

Multifield IMRT (MF-IMRT) has been widely used for the treatment of head-and-neck and pelvic tumors. For whole breast radiotherapy, MF-IMRT could improve dose homogeneity and reduce high-dose areas of ipsilateral lung and heart (for left-side tumors) [6–9]. However, it may result in large low-dose regions in lungs, contralateral breast, and heart that might cause late oncogenesis [10]. Helical TomoTherapy is a new radiation technology that can provide even better dosimetry than MF-IMRT by allowing radiation to be delivered from all gantry angles. But the low-dose region may spread even wider with HT. 

### 4.1. Technical Issues

**Figure 2:**
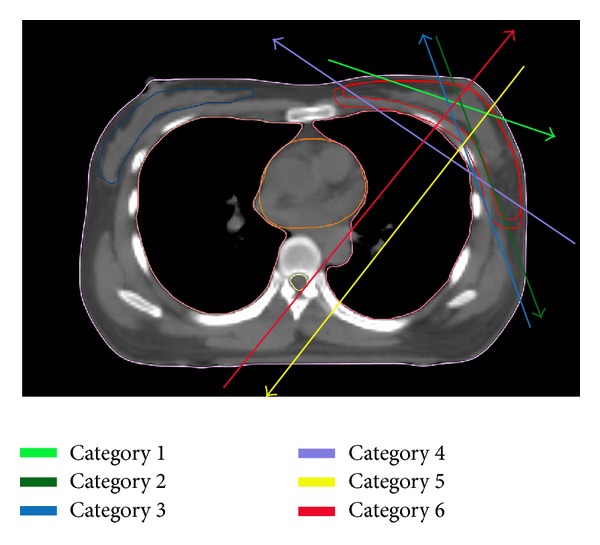
Schematic figure of each category.

In this study, we present a novel HT-based method with similar or even better low-dose area for risk organs. Before the planning process, we divided beamlets delivered from all angles around the patient into six categories (see Figure 2).

- Category 1: beamlets that only pass through PTV. Obviously, these beamlets were most efficient and would cause minimum radiation to the organs at risk, and thus these beamlets were preferred in the planning process.
- Category 2: beamlets that pass through PTV and then through the non-OARs body. These beamlets were less efficient than category 1 but would also cause minimum radiation to the organs at risk.
- Category 3: the opposite of category 2. Beamlets pass through the non-OARs body and then PTV. These beamlets were less efficient than category 2 but were similar to category 2 in that they would not deliver dose to the organs at risk.
- Category 4: beamlets that pass through PTV and organs at risk and then PTV again. This category was common in tangential FIF-IMRT plans and may cause high-dose areas within OARs.
- Category 5: beamlets that pass through PTV and then OARs. These beamlets may be the main cause of “low-dose bath.”
- Category 6: beamlets that pass through OARs and then the PTV. This category is inefficient and should be avoided.

**Figure 3:**
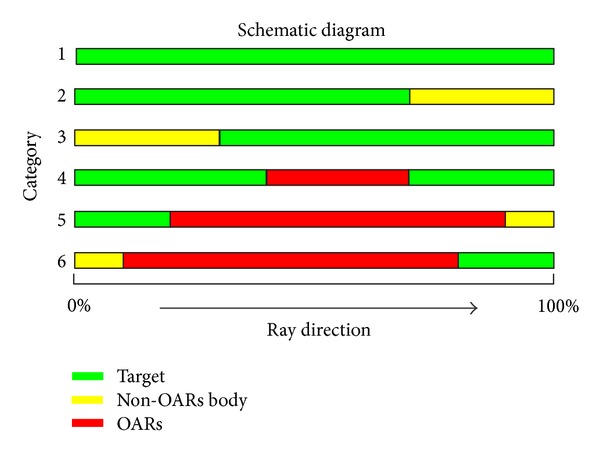
Path of X-ray for every category.

Figure 3 shows the path of X-rays for every categories described above. In the planning process, we tried to design strategies which could raise the proportion of categories 1 and 2 beamlets, reduce the proportion of categories 3 and 4 beamlets, and minimize or even eliminate the category 5 and 6 beamlets.

Given that the breast is situated over the outer border of the chest, if we place beam angles around the chest wall like the peels of an apple, the lungs, contralateral breast, and heart could be spared. The question was how to restrict beams around the chest wall. To prevent dose delivery to a structure, the structure can be designated as “completely blocked” during the planning process. We tried different types of blocks and found this method to be suitable: we draw a tangential line at the posterior border of traditional 3D-CRT beam and define the ipsilateral lung and heart below this line as a new structure “block 1.” If we designate “block 1” as “completely blocked,” and designate contralateral lung and contralateral breast as “directionally blocked,” radiation would be delivered around chest wall as we intend, and these organs or regions would receive less radiation. Obviously, this technique could also be applied for tumors of the right breast.

### 4.2. PTV

Both techniques show adequate PTV coverage. LDC-HT resulted in a lower mean PTV dose than FIF-IMRT. LDC-HT is also associated with a significantly better homogeneity index and conformity index. This supports previous studies that have shown increased homogeneity with HT [11]. However, it is difficult to estimate if these differences would result in clinically detectable benefits such as better cosmesis or a higher local control rate.

### 4.3. Lungs

Radiation-induced lung injury (RILI) variously occurs after whole breast radiotherapy [12]. Most of those cases are subclinical, but about 1–5% are clinically significant radiation pneumonitis [13]. RILI is not only correlated with mean lung dose and *V*_20_; the volume of the lung spared from doses of >5 Gy is an independent dosimetric factor of RILI [14]. Previous studies demonstrated that HT may reduce high-dose regions of ipsilateral lung, but increase the low-dose area [15, 16]. The low-dose “bath” of lung is the main concern of radiation oncologists considering the use of TomoTherapy for whole breast radiation.

In this study, low-dose area constrained helical TomoTherapy-based radiotherapy (LDC-HT) showed dosimetry advantages over FIF-IMRT for ipsilateral lung and heart in not only high-dose levels but also low-dose levels such as *V*_10_ and *V*_5_. For contralateral lung, both techniques can provide good protection, although the mean dose of LDC-HT is higher than FIF-IMRT (0.51 Gy versus 0.09 Gy; *P* = 0.000). So, we expect even lower RILI rates for LDC-HT.

### 4.4. Heart

A recent case-controll study conducted by Darby et al. [17] has highlighted the importance of minimizing incidental cardiac irradiation in patients with breast cancer, as it is a known risk factor for the development of ischaemic heart disease. Importantly, no dose threshold was observed: the increased risk (relative to baseline risk) per Gy of exposure was approximately constant throughout the range of mean radiation doses to the heart studied (from as low as approximately 2 Gy to the maximum >27 Gy). 

In our study, almost all dose levels of heart (*V*_5_ 56.97%, *V*_10_ 58.78%, *V*_20_ 70.70%, *V*_30_ 81.94%, *V*_40_ 91.46%, and *D*_mean_ 44.63%) were significantly decreased with LDC-HT compared with FIF-IMRT. So, we expect lower risk of ischaemic heart disease after LDC-HT whole breast radiotherapy.

### 4.5. Contralateral Breast

LDC-HT increases both *D*_mean_ and *D*_max⁡_ of contralateral breast relative to tangential FIF-IMRT. Although the doses to the contralateral breast are very low with both techniques, the increase in maximal dose and mean dose with LDC-HT might confer an increased risk of secondary breast malignancy, especially in young women [18–21].

Although previous studies demonstrated that improved dosimetry may lead to less toxicity [22, 23], clinical benefits of LDC-HT need further investigation. A randomized phase I/II clinical trial is going on in our centre to evaluate the effect and acute toxicity of LDC-HT over FIF-IMRT.

Finally, LDC-HT had longer treatment time than FIF-IMRT. The use of a 5 cm field width instead of a 2.5 cm field width for TomoTherapy would reduce treatment time with 30–50%, but that might cause worse dosimetry and wider dose spread in the patient superior and inferior to the target. The newly available dynamic jaws capability (TomoEDGE) would eliminate the low superior and inferior doses and should give comparable plans to the 2.5 cm plans [24].

## 5. Conclusions

LDC-HT could have better coverage and dose homogeneity of target volume, better OAR sparing, and even better low-dose areas than 3D-CRT and FIF-IMRT. It could be a feasible method in whole breast radiotherapy. Clinical benefits of LDC-HT need further investigation.

## Figures and Tables

**Table 1:** Average dosimetric characteristics of the target.

	LDC-HT	FIF-IMRT	*P* value
PTV (prescribed dose: 46 Gy)			
*V*_50.6 Gy_ or *V*_110%_	0.7%	15.68%	0.056
*V*_43.7 Gy_ or *V*_95%_	98.73%	98.53%	0.392
Mean dose (Gy)	48.28	49.18	0.002
Maximum dose (Gy)	51.47	51.36	0.667
Homogeneity index	1.08	1.10	0.001
Conformity index	0.83	0.76	0.023

**Table 2:** Average dosimetric characteristics of the organs at risk.

	LDC-HT	FIF-IMRT	*P* value
Ipsilateral lung			
*V*_5 Gy_	21.04	29.16	0.000
*V*_10 Gy_	15.31	21.98	0.000
*V*_20 Gy_	11.58	17.68	0.000
*V*_30 Gy_	7.95	14.65	0.000
*V*_40 Gy_	3.84	9.97	0.000
Mean dose (Gy)	6.12	9.02	0.000
Maximum dose (Gy)	49.98	49.12	0.069
Heart			
*V*_5 Gy_	4.19	9.73	0.000
*V*_10 Gy_	2.75	6.67	0.000
*V*_20 Gy_	1.45	4.96	0.000
*V*_30 Gy_	0.67	3.73	0.000
*V*_40 Gy_	0.20	2.38	0.001
Mean dose (Gy)	1.87	3.37	0.000
Maximum dose (Gy)	42.71	48.33	0.020
Contralateral lung			
Mean dose (Gy)	0.51	0.09	0.000
Maximum dose (Gy)	4.33	1.63	0.000
Both lungs			
*V*_5 Gy_	9.79	13.59	0.000
*V*_10 Gy_	7.44	10.26	0.000
*V*_20 Gy_	5.41	8.24	0.000
*V*_30 Gy_	3.66	6.83	0.000
*V*_40 Gy_	1.79	4.65	0.000
Mean dose (Gy)	3.15	4.25	0.000
Maximum dose (Gy)	49.98	49.12	0.069
Contralateral breast			
Mean dose (Gy)	1.02	0.12	0.000
Maximum dose (Gy)	4.89	1.89	0.000
